# Supplementary material for: Urolithins Modulate the Viability, Autophagy, Apoptosis, and Nephrin Turnover in Podocytes Exposed to High Glucose
Source: Cells. 2022 Aug 9;11(16):2471. doi: 10.3390/cells11162471 (PMC9406555; doi:10.3390/cells11162471)
Supplement: Supplementary file 1 [file cells-11-02471-s001.zip › cells-1819741-supplementary.pdf]

Material S1. HPLC system and conditions of separations

The Shimadzu HPLC system (Kyoto, Japan) consisting of: two pumps LC-20AD, a degasser DGU-20A5, a semi-micro mixer, an autosampler SIL20AC $_{XR}$, a column oven CTO-20AC, a diode array detector SPD-M20A, a controller CBM-20A, a valve unit FCV-20AH$_2$, a nitrogen generator PEAK Scientific GeniusXE35 230. Conditions of separation: Kinetex ® 2.6 µm C18 100 Å column (100 mm x 2.1 mm) (Phenomenex, .Baltimore, USA), column temp. 25°C. The gradient elution program is presented in table 1, the following solvents were used: solvent A water :formic acid (100:0.1 *v/v*) and solvent B water: acetonitrile: formic acid (50:50: 0.1, *v/v/v*). Flow rate of mobile phase 0.3 mL/min., UV detection – 305 and 350 nm.

Supplementary Table S1 Gradient elution program

Time (min)	%B in A+B
0	10
7	36
17	56
22	100
30	100
31	10
45.01	10

Material S2. Validation of the developed HPLC-MS/MS method for the determination of urolithin A and its metabolite.

The method was validated by determining the linearity, limit of quantitation (LOQ), limit of detection (LOD), intra- and inter- day repeatability and extraction recovery of urolithin A. Calibration curves were measured for urolithin A as a constituent of two matrix types: NG medium (normal glucose level) and HG medium (high glucose level) by diluting urolithin A at concentrations from 1 µM to 12 µM in cell culture medium (injection volume was 1 µL). The experiment was carried out in triplicate. The limit of detection (LOD) and quantification (LOQ) were estimated by S/N ratio 3 and 10, respectively. The linearity for the working concentrations of urolithin A was assessed by determining the correlation coefficient (Supplementary table 2).

Supplementary Table S2. The values of validation parameters for urolithin A (regression equations, R2, LOQ and LOD)
determination in cell culture media with normal (NG) and high (HG) glucose level.

Type of analyzed matrix	Range of UA concentration (µM)	Regression equation	R^2	LOD	LOQ
HG cell culture medium	1-12	y=7711.2x-1360.7	0.993	0.2	0.67
NG cell culture medium	1-12	y=7316.7x-2995.4	0.992	0.27	0.905

Urolithin A recovery was assessed by adding the compound at a concentration of 10 µM to the HG and NG medium, followed by inoculation of podocytes. Urolithin A was extracted from the cell culture medium immediately after inoculation as described in Preparation of samples for analysis paragraph. Three samples were examined for each type medium. The determined recovery of the Urolithin A was 72.2% for the NG cell culture medium and 77.1 % for the HG cell culture medium.

The intra- and inter-day repeatability of the developed method was evaluated by analyzing continuous injections of the same sample six times a day and for three consecutive days, and was expressed as relative standard deviation

(RSD). The intra-day precision of Urolithin A determination was range from 4.65% and the inter-day precision was 5.97%.

Supplementary Table S3. Primary antibodies.

Primary Antibody	Application and Dilution	Catalog number	Source
Nephrin (G-8)	FC 1:200, IF 1:50	sc-376522	Santa Cruz Biotechnology, U.S.
Nephrin	FC 1:100, WB 1:500	ab216341	Abcam, Cambridge, UK
EEA-1	IF 1:100	MA5-14794	Invitrogen, Thermo Fisher Scientific, U.S.
Beta Actin	WB 1:5000	ab8227	Abcam, Cambridge, UK
SQSTM1 / p62	WB 1:10 000	ab109012	Abcam, Cambridge, UK
APG5L/ATG5	WB 1: 1000	ab228668	Abcam, Cambridge, UK
LC3B	WB 1:2 500	ab51520	Abcam, Cambridge, UK
Bcl-2	WB 1:1000	ab196495	Abcam, Cambridge, UK
HRP Alpha 1 Sodium Potassium ATPase	WB 1:5000	ab196696	Abcam, Cambridge, UK
Rabbit IgG Isotype Control	IF 1:100, FC 1:100	10500C	Invitrogen, Thermo Fisher Scientific, U.S.

WB: Western blot, **FC**: Flow Cytometry, **IF**: Immunofluorescence, **EEA-**1: Early Endosome Antigen 1, **SQSTM1 /p62**: Sequestosome 1/ubiquitin-binding protein p62, **APG5L/ATG5**: Autophagy related 5 protein, **LC3B**: microtubule-associated protein 1A/1B light chain 3B, **Bcl-2**: B-cell lymphoma 2 protein, **HRP**: Horseradish peroxidase

Supplementary Table S4. Secondary antibodies.

Secondary Antibody	Application and Dilution	Catalog number	Source
Goat anti-rabbit/Alexa Fluor 488	FC 1:200 IF 1:100	A32731	Invitrogen, Thermo Fisher Scientific, U.S.
Goat anti-rabbit/Alexa Fluor 555	IF 1:100	A-21429	Invitrogen, Thermo Fisher Scientific, U.S.
Goat anti-rabbit/Alexa Fluor 647	IF 1:100	ab150079	Abcam, Cambridge, UK
MFP™-DY-490-Phalloidin	IF 1:100	MFP-D490-33	MoBiTec Molecular Biotechnology, Germany
Anti-rabbit IgG, HRP-linked	WB 1: 2000	7074S	Cell Signaling Technology, U.S.

WB: Western blot, **FC**: Flow Cytometry, **IF**: Immunofluorescence, **HRP-linked**: Horseradish peroxidase-linked
